# Solexa Sequencing Identification of Conserved and Novel microRNAs in Backfat of Large White and Chinese Meishan Pigs

**DOI:** 10.1371/journal.pone.0031426

**Published:** 2012-02-15

**Authors:** Chen Chen, Bing Deng, Mu Qiao, Rong Zheng, Jin Chai, Yi Ding, Jian Peng, Siwen Jiang

**Affiliations:** 1 Key Laboratory of Swine Genetics and Breeding of Agricultural Ministry, and Key Laboratory of Agricultural Animal Genetics, Breeding and Reproduction of Ministry of Education, College of Animal Science and Technology, Huazhong Agricultural University, Wuhan, People's Republic of China; 2 Department of Animal Nutrition and Feed Science, College of Animal Science and Technology, Huazhong Agricultural University, Wuhan, People's Republic of China; University of Barcelona, Spain

## Abstract

The domestic pig (*Sus scrofa*), an important species in animal production industry, is a right model for studying adipogenesis and fat deposition. In order to expand the repertoire of porcine miRNAs and further explore potential regulatory miRNAs which have influence on adipogenesis, high-throughput Solexa sequencing approach was adopted to identify miRNAs in backfat of Large White (lean type pig) and Meishan pigs (Chinese indigenous fatty pig). We identified 215 unique miRNAs comprising 75 known pre-miRNAs, of which 49 miRNA*s were first identified in our study, 73 miRNAs were overlapped in both libraries, and 140 were novelly predicted miRNAs, and 215 unique miRNAs were collectively corresponding to 235 independent genomic loci. Furthermore, we analyzed the sequence variations, seed edits and phylogenetic development of the miRNAs. 17 miRNAs were widely conserved from vertebrates to invertebrates, suggesting that these miRNAs may serve as potential evolutional biomarkers. 9 conserved miRNAs with significantly differential expressions were determined. The expression of miR-215, miR-135, miR-224 and miR-146b was higher in Large White pigs, opposite to the patterns shown by miR-1a, miR-133a, miR-122, miR-204 and miR-183. Almost all novel miRNAs could be considered pig-specific except ssc-miR-1343, miR-2320, miR-2326, miR-2411 and miR-2483 which had homologs in *Bos taurus*, among which ssc-miR-1343, miR-2320, miR-2411 and miR-2483 were validated in backfat tissue by stem-loop qPCR. Our results displayed a high level of concordance between the qPCR and Solexa sequencing method in 9 of 10 miRNAs comparisons except for miR-1a. Moreover, we found 2 miRNAs, miR-135 and miR-183, may exert impacts on porcine backfat development through WNT signaling pathway. In conclusion, our research develops porcine miRNAs and should be beneficial to study the adipogenesis and fat deposition of different pig breeds based on miRNAs.

## Introduction

MicroRNAs (miRNAs), the small non-coding RNAs (typically 19–23 nucleotides), were firstly discovered in *C. elegans* [1] and play important roles in regulating post-transcriptional translation [2]. These small non-coding RNAs extensively exist in the cells of plants, animals and some viruses [3]–[5]. Increasing evidence demonstrates that miRNAs have evolved as a major class of gene-regulatory molecules critical for diverse biological processes, such as development, differentiation, proliferation and diabetes in eukaryotes [6]–[9].

miRNAs are initially transcribed from miRNA genes located mainly in the intergenic genomic region. By RNA polymerase II, they yield transcripts called primary miRNAs (pri-miRNAs), which are processed to generate miRNA precursors (pre-miRNAs) by a complex containing Drosha and then by Dicer to excise miRNA:miRNA-star (miR:miR*) duplexes. Of which one strand is more stable and preferentially incorporated into an RNA-induced silencing complex (RISC), while the other strand (miRNA*) is usually discarded [10]. The miRNA then guides the RISC to bind the UTRs and CDS of target mRNAs, where it downregulates the gene expression, often by blocking protein production or by degrading the target mRNA [11], [12].

The pig (*Sus scrofa*), an important species in animal production industry, is a right model for studying adipogenesis and fat deposition. Increasing evidences suggest that miRNAs contribute to the regulation of fat development [13]–[15]. However, rare reports could be read about the porcine miRNAs that effect the adipocyte differentiation and adipogenesis. The total number of porcine miRNA genes discovered by human beings so far is much lower, which affects the study on pigs. Therefore, finding more miRNAs in pigs can be contribute to various and further studies on pigs. Some recent articles have depicted miRNAs in pigs [16]–[19], but most of these researches concentrated on miRNAs of porcine skeletal muscle. Backfat is another important tissue, however, there have been scarce reports on miRNAs identified from this tissue.

Solexa sequencing technology is a convincing strategy for identifying miRNAs and has been utilized by several laboratories to identify miRNAs [10], [20], [21]. To enrich the repertoire of miRNAs in pigs, we constructed and sequenced two small RNA libraries prepared from the backfat tissue of 150-day-old Large White (LW) and Meishan (MS) pigs. The former is a lean type pig, and the latter is a Chinese indigenous fatty pig. As the spatial distribution of miRNAs may contribute to the mechanistic understanding of adipocyte differentiation and adipogenesis, this study intended to provide deeper insights into dynamic regulation of the conserved and novel miRNA levels during different breeds of pigs.

## Results and Discussion

### Solexa sequencing data between the two small RNA libraries

Small RNAs (18–30 nt) were obtained from the total RNA, 5′ and 3′ RNA adaptors were ligated to the RNA pool, and the adaptor-ligated small RNAs were then subsequently subjected to RT-PCR to produce sequencing libraries. PCR products were purified and small RNA libraries were sequenced using Solexa. Primary analysis was performed on the 35 nt tags. First, we got rid of the 5′ adaptor, the low quality tags to obtain clean tags. The length distribution of the clean tags was subsequently summarized. Then, the clean tags were annotated into different categories, and finally those tags which couldn't be annotated to any category were used to predict the novel miRNAs. The work flow of Solexa sequencing is shown in Figure S1. The flow results of raw sequence data for the two libraries are shown in Table S1. The clean reads in the small RNA library of Large White was more than 19 million, and it was the same for Meishan small RNA library. Small RNA annotation is presented in Table S1, A-B. The distribution of sequence lengths was similar between both small RNA libraries by following the law of normal distribution, and the majority of sequences were 22 nt in length (Table S1, C–D). miRNA had a much tighter length distribution, centering on 21–24 nt. The number of 21–23 nt sequences was significantly greater than those shorter or longer sequences, and almost half of the sequences in Large White (49.79%) and Meishan (46.28%) are canonical 22 nt miRNA, which coincided with the known specificity for Dicer processing and the features of mature miRNAs [22], [23]. Nearly 10 million reads of Large White and Meishan small RNA libraries were respectively screened as miRNA candidates and used for further analysis. The common and specific tags of two libraries, including the summary of unique tags and total tags were listed in Table S1, E in which the total number of unique sRNAs reads in Large White and Meishan small RNA libraries was almost 0.38 million and 0.49 million, respectively. These unique sRNAs sequences contained 0.1 million common sequences between the two libraries. The common sequences of total sRNAs between the two libraries were much higher (accounted for 97.75%) than that of unique sRNAs (accounted for 13.63%), this indicated that the number of Large White-specific and Meishan-specific sequences was much smaller than that of common sequences between the two libraries.

### Identification of conserved porcine miRNAs

After successive filtering of these data sets, we identified 215 unique miRNAs comprising 75 pre-miRNAs, of which 49 miRNA*s were first identified, 73 miRNAs were overlapped in both libraries, and 140 were novel predicted miRNAs and 215 unique miRNAs were collectively corresponding to 235 independent genomic loci (Table S2). However, 13 miRNA/miRNA* were detected in only one small RNA library. For example, ssc-miR-153, miR-325, miR-135-1*, miR-135-2*, miR-146b*, miR-15a*, miR-215* and miR-323* were only identified in Large White, contrary to the patterns displayed by ssc-miR-101a-1*, miR-103*, miR-183*, miR-1a* and miR-210*, indicating that these miRNAs may function in the physiology or development of the backfat tissue.

**Figure 1:**
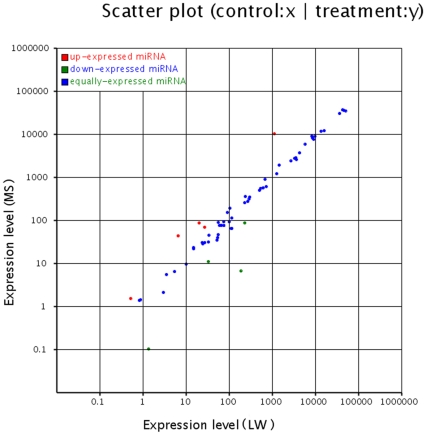
The differentital expressions of porcine conversed miRNAs between Large White and Meishan were shown. Each point in the figure represents a miRNA. Red points represent miRNAs with fold change>2, blue points represent miRNAs with 1/2<fold change ≤2, green points represent miRNAs with fold change ≤1/2.

The known miRNAs expression profiles between two libraries are shown in Table 1. The expression of the known miRNAs in the two samples was demonstrated by plotting Log2-ratio and Scatter Plot (Figure 1). The results showed that 9 miRNAs were significantly different between the two libraries, such as the expression of miR-215, miR-135 and miR-146b was higher in Large White pigs, opposite to the patterns shown by miR-1a, miR-133a, miR-122, miR-204 and miR-183 (Table 1), suggesting that these miRNAs may have effects on the development of backfat tissue. Detailed information of known miRNAs in both libraries, including sequence reads and hairpin structures are shown in Table S3. Lots of miRNAs were found to have end variants, differing by one or several nucleotides most commonly in the 3′ end. For example, miR-204 and miR-450 only had 3′ end variants. miR-107, miR-122 and miR-19a were respectively differed by only one nucleotide in the 5′ end, but they had several 3′ end variants. Such variants may come from altered miRNA processing, preferential degradation at the miRNA ends, or post-transcriptional modifications, including RNA editing [24], [25].

**Table 1:** The expression profile of all known miRNAs in Large White and Meishan small RNA libraries.

miRNA-name	LW expressed	MS expressed	LW-std	MS-std	fold change(log2 MS/LW)	p-value	sig -lable
ssc-let-7c	877585	702481	45536.7915	35816.4719	−0.34640941	0	
ssc-let-7f	821249	708567	42613.5867	36126.7708	−0.23824517	0	
ssc-let-7i	52800	46207	2739.7262	2355.8953	−0.2177563	2.1299E-124	
ssc-miR-101a	113825	113897	5906.2373	5807.1161	−0.02441744	5.38434E-05	
ssc-miR-103	191974	172479	9961.2915	8793.9592	−0.17981996	0	
ssc-miR-106a	472	554	24.4915	28.2461	0.20577055	0.022686026	
ssc-miR-107	652	872	33.8315	44.4595	0.39412458	1.1522E-07	
ssc-miR-122	392	1678	20.3404	85.554	2.07248726	3.8728E-184	**
ssc-miR-125b	70520	50788	3659.1949	2589.4607	−0.4988746	0	
ssc-miR-128	529	597	27.4492	30.4385	0.14913316	0.083386279	
ssc-miR-130a	2138	1264	110.9382	64.4459	−0.78359575	1.06424E-54	
ssc-miR-133a	126	849	6.538	43.2868	2.72700587	8.7333E-130	**
ssc-miR-135	26	2	1.3491	0.102	−3.72535622	1.3011E-06	**
ssc-miR-136	293	423	15.2034	21.5669	0.50442482	3.59769E-06	
ssc-miR-139	1158	1478	60.0872	75.3568	0.32668001	7.08989E-09	
ssc-miR-140	11884	11133	616.6459	567.6236	−0.11950771	3.3378E-10	
ssc-miR-140*	177156	146169	9192.404	7452.526	−0.30271271	0	
ssc-miR-145	5863	6830	304.2238	348.2322	0.19491659	2.99995E-14	
ssc-miR-146b	4469	1714	231.8908	87.3895	−1.40791374	4.1842E-289	**
ssc-miR-148a	969529	683978	50307.651	34873.0839	−0.52866387	0	
ssc-miR-15a	5238	5325	271.7933	271.4987	−0.0015646	0.955478867	
ssc-miR-15b	1074	893	55.7285	45.5302	−0.29159152	7.74903E-06	
ssc-miR-16	24506	23734	1271.5858	1210.0941	−0.07150957	5.23792E-08	
ssc-miR-17	5703	6089	295.9216	310.4518	0.06915429	0.009280647	
ssc-miR-18	287	452	14.8921	23.0455	0.62993786	4.84017E-09	
ssc-miR-181a	83735	71712	4344.9048	3656.2851	−0.248946	6.3469E-253	
ssc-miR-181b	14048	11933	728.9332	608.4121	−0.26073777	6.49187E-48	
ssc-miR-181c	1055	779	54.7426	39.7178	−0.46287825	8.46657E-12	
ssc-miR-183	519	1342	26.9303	68.4228	1.34524684	8.87706E-81	**
ssc-miR-184	2248	1273	116.6459	64.9048	−0.84573852	3.25294E-65	
ssc-miR-185	4448	4947	230.8012	252.2262	0.12806742	1.72089E-05	
ssc-miR-186	28047	37058	1455.3239	1889.4274	0.37660881	9.0493E-241	
ssc-miR-196	1747	2974	90.6497	151.6314	0.74219439	1.69018E-67	
ssc-miR-199b*	261685	229155	13578.5084	11683.6237	−0.2168372	0	
ssc-miR-19a	196	188	10.1702	9.5853	−0.08545256	0.561914591	
ssc-miR-1a	21904	198062	1136.5713	10098.3259	3.15135605	0	**
ssc-miR-20	13192	17233	684.5164	878.6362	0.36018084	3.7558E-104	
ssc-miR-204	10	30	0.5189	1.5296	1.55962599	0.00175365	**
ssc-miR-205	68	108	3.5284	5.5065	0.64212149	0.003710943	
ssc-miR-21	710609	587666	36872.615	29962.551	−0.29938928	0	
ssc-miR-210	106	125	5.5002	6.3732	0.21253385	0.265328125	
ssc-miR-214	2218	2191	115.0893	111.7096	−0.04300053	0.322383625	
ssc-miR-215	3618	128	187.7335	6.5262	−4.84629903	0	**
ssc-miR-216	17	28	0.8821	1.4276	0.69457768	0.117088654	
ssc-miR-217	58	41	3.0095	2.0904	−0.52574479	0.073374517	
ssc-miR-221	1931	1784	100.1972	90.9585	−0.13956182	0.00321048	
ssc-miR-224	638	215	33.105	10.9619	−1.59455124	3.44467E-51	**
ssc-miR-23a	9821	9749	509.5994	497.0594	−0.0359453	0.081373414	
ssc-miR-24	159722	177278	8287.7754	9038.6395	0.12512072	1.2767E-139	
ssc-miR-26a	166868	160083	8658.5725	8161.9407	−0.08521697	5.70634E-64	
ssc-miR-27a	68858	54931	3572.9558	2800.6944	−0.3513335	0	
ssc-miR-29b	1093	1727	56.7144	88.0523	0.6346456	1.00582E-30	
ssc-miR-29c	2009	3691	104.2445	188.1881	0.85220413	3.1889E-105	
ssc-miR-30a	311295	234230	16152.7094	11942.376	−0.43568628	0	
ssc-miR-30b	4621	6932	239.7779	353.4327	0.55973683	1.41568E-94	
ssc-miR-30c	10323	10848	535.6476	553.0927	0.04623713	0.01975291	
ssc-miR-32	1273	1498	66.0544	76.3765	0.20947414	0.000136849	
ssc-miR-326	16	27	0.8302	1.3766	0.72957858	0.108360144	
ssc-miR-34a	1446	1807	75.0311	92.1311	0.29619953	5.39272E-09	
ssc-miR-450	1018	679	52.8228	34.6193	−0.60958423	7.00986E-18	
ssc-miR-503	647	609	33.572	31.0503	−0.11265128	0.166634817	
ssc-miR-7	463	595	24.0245	30.3365	0.33654824	0.000159445	
ssc-miR-9-1	1480	1459	76.7954	74.3881	−0.04594805	0.387961957	
ssc-miR-9-2	1480	1459	76.7954	74.3881	−0.04594805	0.387961957	
ssc-miR-95	1295	1502	67.1959	76.5805	0.18860388	0.000558833	
ssc-miR-99b	64674	53630	3355.8532	2734.3621	−0.2954753	1.1759E-270	

**Note:** (1) LW-std: The result of Solexa sequencing exhibited, presented the normalized expression level of miRNA in Large White library. (2) MS-std: The result of Solexa sequencing exhibited, presented the normalized expression level of miRNA in Meishan library. (3) fold-change(log2 MS-std/LW-std): Fold change of miRNAs in both samples. (4) p-value: p value manifests the significance of miRNA differential between two samples. Less p value indicates more significance of difference of miRNA. (5) sig-lable: significance label.

*: fold change(log2)>1 or fold change(log2)<−1, and 0.01≤p-value<0.05.

**: fold change(log2)>1 or fold change(log2)<−1, and p-value<0.01. None: Others.

miR-1a and miR-133a had been reported to be exclusively expressed in muscle [7]. In our analysis, we identified that they also expressed in porcine backfat tissue, the reads number of miR-1a and miR-133a was 0.2 million and 1028, respectively, indicating that these two miRNAs may be effective in adipogenesis. A number of miRNAs (miR-136, miR-140, miR-204, miR-221, and miR-325) originated from the 5′ and 3′ arms of the miRNA hairpin precursors, exhibited a similar number of sequence reads. The approximate equivalent expression rates of miRNA and miRNA* (miR-136:293, miR-136*:107 in Large White library) may be due to the similar 5′ end stability which results to equal incorporation of either strand into the RISC and protection from degradation [26]. Accordingly, this miRNA precursor may produce functional molecules on both arms.

### Identification of novel porcine miRNAs

Base on the Solexa sequencing, we identified 140 novel porcine miRNAs, which corresponding to 156 genomic loci. 87 in Large White pigs, 102 novel miRNAs in Meishan pigs. Their secondary structures are shown respectively in Table S4. 2 of the 156 genomic loci were located in the exons of protein-coding genes, 35 in introns, 11 in the introns and exons of single protein-coding genes, 3 in the introns and exons of two protein-coding genes, others were in intergenic regions (Table S2). Almost all these miRNAs could be considered pig-specific except ssc-miR-1343, miR-2320, miR-2326, miR-2411 and miR-2483 which had homologs in *Bos taurus* (Table 2).

**Table 2:** Comparison of predicted novel miRNAs in pigs with their homologs in *Bos taurus*.

miRNA-name	precusor-miRNA	Minimum free energy(kcal/mol)
bta-mir-1343	GGCTTCGGTGCTGGGGAGCGGCCCCCGGGCGGGCCTCTGCTCTGGCCC**CTCCTGGGGCCCGCACTCT**CGCTCCGGGCC	
ssc-mir-1343	GGCTTCGGTGCTGGGGAGCGGCCCCCGGGCGGGCCTCTGCTCTGGCCC**CTCCTGGGGCCCGCACTCTCGC**TCCGGGTC	−50.3
bta-mir-2320	TGTCCCCA**TGGCACAGGGTCCAGCTGTCGGC**CGTGATACCCGATGGGTCGATGATGGTCCCTGTGTTTTGGGGCG	
ssc-mir-2320	TGTCCCCA**TGGCACAGGGTCCAGCTGTCGG**CTGTAATACCCGATGGGTCGATGATGGTCCCTGTGTTTGGGGCG	−37.7
bta-mir-2366	CCCCGGGGTCCTCTTGTCTGAGCCCCAGAAAGAGGAGAGAGTGC**TGGGTCACAGAAGAGGGTCTGG**GGG	
ssc-mir-2366	CCCCAGGGTCCTCTTGTCTGAGCCCCAGAAAGAGGAGAGAGCGC**TGGGTCACAGAAGAGGGTCTGG**GGG	−31.6
bta-mir-2411	GAGGAAATG**TGGAGTGACTGTCAGATGCAGCCA**GCAGAATAAGTGGTTTGGCTGAACTGTCTTACTCCCACATCCTC	
ssc-mir-2411	GAGGCAATGT**GGAGTGACTGTCAGATGCAG**CCATCAGAATAGGTGATTTGGCTGAACTGTCATACTCCCACATCCTC	−29.2
bta-mir-2483	GAGTGAAAAGTTCCGTCAACCATCCAGCTGTTTGAGGTGATGC**AAACAAACATCTGGTTGGTTGAGAGA**ATTTTTTACTT	
ssc-mir-2483	GAGTGAAAAGTTCCGTCAACCATCCAGCTGTTTGGGGTGATGC**AAACAAACATCTGGTTGGTTGAGAGA**ATTTTTTACTT	−37.3

**Note:** The bold are the mature miRNA sequences.

The read number for each novel miRNA was much lower than that for the majority of conserved miRNAs. For instance, ssc-miR-2411 and ssc-un0012, both were novel miRNAs, the total reads were only 131 and 5, respectively. However, the total reads of two conserved miRNAs (ssc-miR-148a and ssc-let-7c) were striking, almost 1.7 million and 1.6 million, respectively (Table S2). This might suggest a specific role for these novel miRNAs during developmental stages. Since this study investigated the miRNAs of backfat tissue from female Large White and Meishan pigs, whether these low-abundant miRNAs are expressed at higher levels in other tissues and organs remains to be investigated. Future experiments could provide more insight into the function of these miRNAs.

### Validation of porcine miRNAs

**Figure 2:**
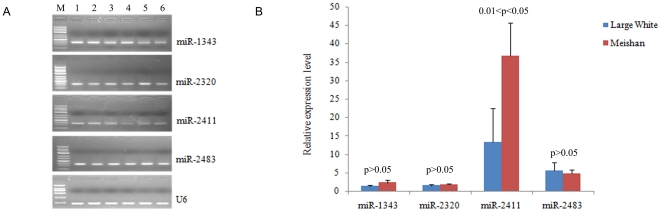
Four novel miRNAs were cloned and validated in porcine backfat tissue by stem-loop qPCR. A. miR-1343, miR-2320, miR-2411 and miR-2483 were cloned in porcine backfat tissue of Large White and Meishan samples. M represents DNA size marker, DL700 and DL2000. Large White samples (lane 1–3) and Meishan (lane 4–6) samples. U6 snRNA was used as an internal control. A 3% agarose gel, stained by ethidium bromide, was run to indicate the cloned miRNA because of the small product size, and the 1.5% agarose gel was used to indicate U6. B. The four novel miRNAs were validated by stem-loop qPCR. All experiments were performed three times. The data shown in Y-axis were calculated using the expression values of 2 ^−ΔΔCt^ and expressed as means ± standard error. The significance of differences for the expression between samples was calculated by one-way ANOVA. The corresponding significance value (p) was listed above their respective columns. 0.01<p<0.05 was considered to be significant.

**Figure 3:**
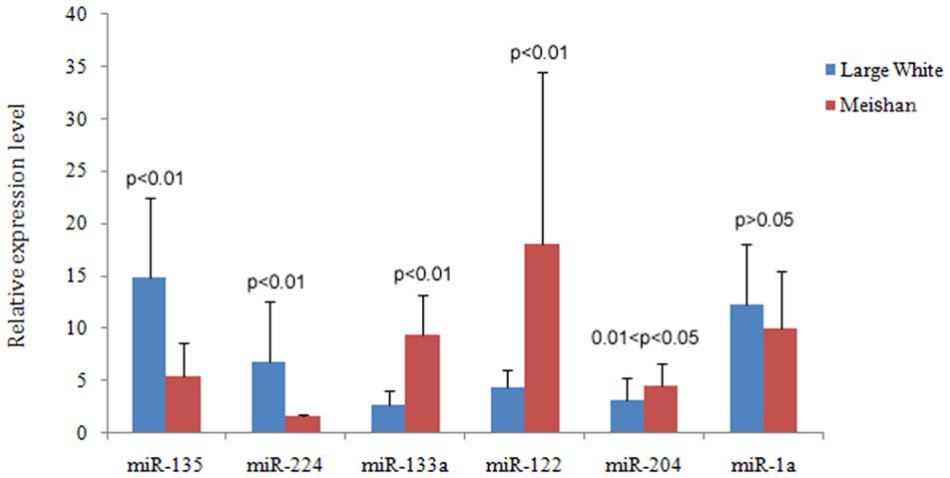
Validation of the miRNAs with significantly differential expressions using stem-loop qPCR method. Expression levels of 6 miRNAs were detected by Real-time PCR. The number of biological replicates is three. The corresponding significance value (p) was shown above their respective columns. 0.01<p<0.05 was considered to be significant and p<0.01 significantly different.

We adopted stem-loop qPCR to validate our sequencing data [27]. A total of 4 novel miRNAs were cloned and validated in backfat tissue by qPCR, and they all could express (Figure 2, A). The expressions of miR-1343, miR-2320 and miR-2411 were slightly higher in Meishan samples; miR-2483 higher in large White samples (Table S2). And our qPCR results showed the consistency (Figure 2, B). In addition, the qPCR results suggested that 6 miRNAs which were sequenced by Solexa and displayed significantly differential expressions were expressed in porcine backfat tissue, but the expression levels of different miRNAs varied (Figure 3). In general, the results of qPCR validated the sequencing results and they were consistent. The expression of miR-135, miR-224 was significantly higher in Large White pigs, opposite to the expression of miR-133a, miR-122. The expression of miR-204, higher in Meishan pigs, was significant between the two samples (Figure 3). However, miR-1a was an exception, its expression level was higher in Large White pigs, which was inconsistent with the sequencing results, and no significant difference was observed. The discrepancy in the miRNA expression may because of differently adopted methods, such as cloning protocols, and various reads numbers that could be produced. Our results displayed a high level of concordance between the two methods in the 9 of 10 miRNAs comparisons, showing that the majority of our miRNA expression data embody the actual miRNA expression levels.

### Seed edit of miRNAs and clusters of miRNA sequences

The position of 2∼8 of a mature miRNA is called seed region which is highly conserved, and the seed region most often binds to a target site in the 3′ UTR of the target mRNA by perfect complementarity. The target of a miRNA might be different with the change of nucleotides in this region and any nucleotide difference in seed region may lead to the dysfunction of miRNA and then resulted in the variation of phenotypic characteristics [28], [29].

In our analysis, miRNAs which might have seed edit can be distinguished by matching unannotated sRNA with porcine mature miRNAs from miRBase15.0, allowing a mismatch on certain position. We found in the two samples a total of 22 mature miRNAs with single nucleotide substitution in seed region (Table S5). Our study demonstrats that A -to- G, G -to- A, C -to- T, G -to- T, A -to- C and T -to- C were the dominant substitutions, which is consistent with published reports [30], [31]. In our research, small RNA sequences not perfectly aligning to the genome were often discarded as mismatched sequences, which led to ‘sequencing errors’. However, RNA editing or post-transcriptional modifications, not absolutely technical artifacts, could also be attributed to ‘sequence errors’ [30]. Obviously, high-abundant miRNAs (let-7c, let-7f, miR-148a, miR-21 and miR-24) had higher edited probability in backfat tissue. This indicates that highly-expressed miRNAs targeted more genes in this tissue. Accordingly, nucleotide changes in seed region could increase miRNA diversity, change their targets and may have important phenotypic consequences.

Of the conserved miRNAs, 66 miRNAs, almost occupy one-third of total amount, were also found in previous report [32]. However, the remaining miRNAs are distinct, this may be due to the different porcine breeds and developmental stages. Several miRNAs such as let-7, miR-27 and miR-103 are reported to perform very important functions in adipogenesis [33]–[35]. Moreover, published report showed that miR-196, miR-214, miR-199b, miR-186, miR-101 and miR-27a were related to bovine backfat thickness [36]. These miRNAs were also observed in our miRNAs expression patterns, suggesting that they may exert impacts similar to those of their orthologs on porcine adipogenesis and adipose tissue.

**Figure 4:**
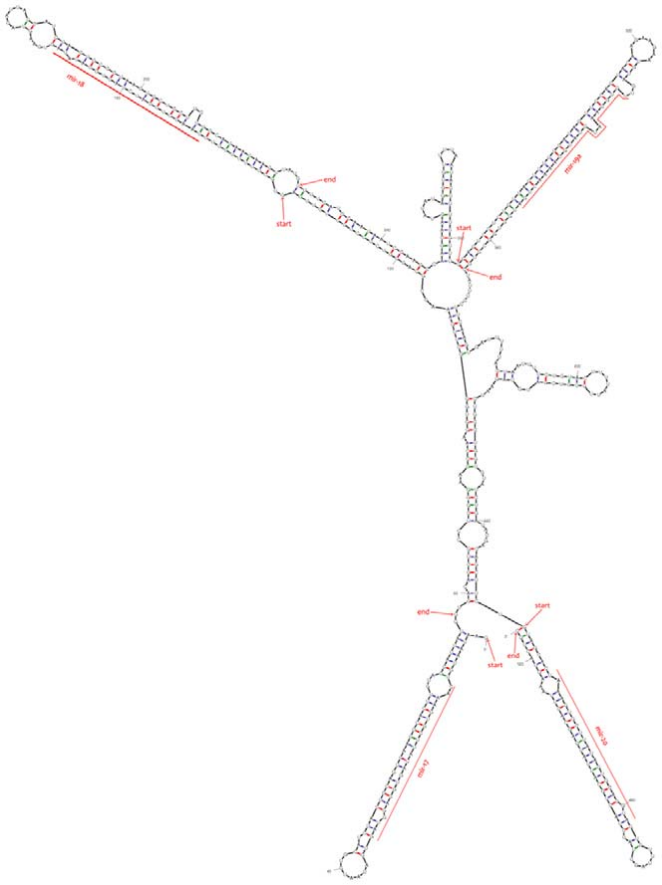
The folding secondary structure of porcine mir-17-mir-20 cluster predicted computationally by RNAfold. Folding secondary structure of porcine mir-17 cluster including four miRNAs (mir-17, mir-18, mir-19a and mir-20) and flanking sequences was predicted by RNAfold. The sequences from start to end represent pre-miRNAs.

The 215 miRNAs were categorized into clusters on the basis of their locations on chromosomes (miRNA-miRNA distance<10 kb). In brief, 18 miRNAs (13 conserved miRNAs and 5 predicted miRNAs) were found to be in 7 different clusters (Table S6). The mir-17-mir-20 cluster consisted of four miRNA genes. By using RNAfold [37], its folding structure was predicted (Figure 4), which was consistent with the previous report in human [38]. Such clustered miRNAs could be coordinately transcribed together as polycistronic transcripts.

**Figure 5:**
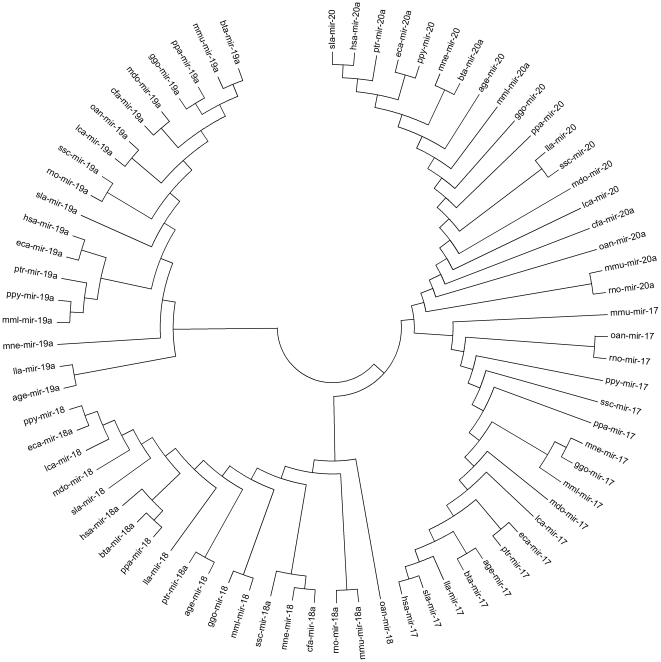
Phylogenetic trees of mir-17-mir-20 cluster among different mammalian species were constructed using MEGA4.

We implemented the phylogenetic analysis of mir-17 cluster among different mammalian species using MEGA4 [39]. mir-17 and mir-20 had closer phylogenetic and evolutionary relationship with each other (Figure 5). The result partially proved that mir-20 resulted from duplication of mir-17 and the history of mir-17 cluster might be closely linked to the early evolution of the mammalian lineage [38]. Furthermore, we identified that 17 miRNAs were extensively conserved from vertebrates to invertebrates (Table S7), suggesting that these miRNAs may serve as potential evolutional biomarkers.

### Potential regulation pathway of miRNA in porcine adipogenesis

In the present study, the fact that miR-122 expressed in backfat is inconsistent with current report that this miRNA is liver-specific [40]. Researches have shown that miR-122 regulates the pathway of cholesterol biosynthesis, promotes lipid synthesis and inhibits fatty-acid oxidation in the adult mouse liver. In the present study, several key genes known to regulate fatty-acid synthesis and oxidation were downregulated, including *FASN* and *ACC* after miR-122 inhibition and their fatty-acid synthesis rate was also reduced [14]. We therefore could postulate that miR-122 may influence lipid metabolism and adipogenesis in the above-mentioned way in backfat tissue. However, the fine-tuned mechanism is worthy of further research.

**Figure 6:**
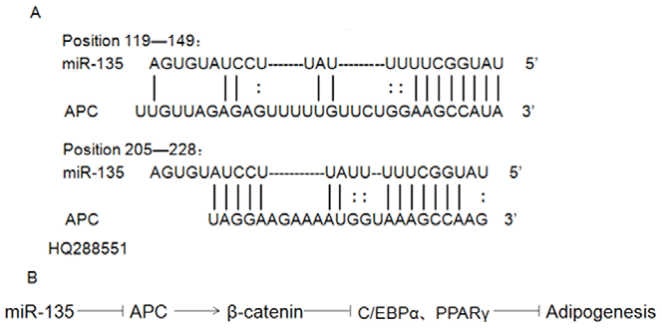
Hypothesis of miR-135 in porcine adipogenesis. A. 3′ UTR of *APC* was predicted as a potential target of miR-135 (position and GenBank No. are listed). B. Potential regulation pathway of miR-135 in porcine adipogenesis.

Recently, a novel mechanism for *APC* regulation has been found. It was shown that miR-135 targets the 3′ UTR of *APC* and suppresses its expression, then *APC*-free β-catenin stimulates the WNT signaling pathway, leading to active transcription of target genes, and induce downstream WNT pathway activity [41]. Previous report has demonstrated that WNT signaling pathway inhibits adipocyte differentiation by blocking the expression of *C/EBPα* and *PPARγ*, two transcription factors indispensable for adipogenesis [42]. So, we could speculate that miR-135 may suppress porcine adipogenesis through activating WNT signaling pathway by targeting *APC* (Figure 6).

**Figure 7:**
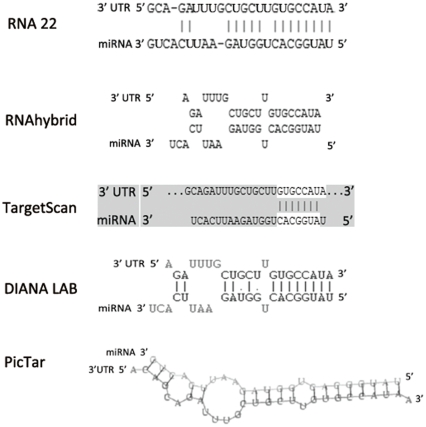
Potential miR-183-*LRP6* interaction was predicted by algorithms RNA22, RNAhybrid, TargetScan, DIANA LAB and PicTar. We found putative binding site in 3′ UTR of *LRP6*, and the predicted results were identical among five different algorithms.

To explore the potential function of miRNAs with significantly differential expression in backfat tissue, we adopted algorithms RNA22, RNAhybrid, TargetScan, DIANA LAB and PicTar to predict the targets of miRNAs. The predictions were conducted according to the interactions of human mRNA-miRNA because of the absence of porcine miRNAs in current versions of the above-mentioned algorithms. We found *LRP6*, the surface co-receptor component of WNT signaling pathway, had putative binding site in its 3′ UTR (Figure 7), and this indicated that *LRP6* may be the potential target of miR-183. Previous research demonstrated that LRP6 co-receptor is critical for regulation of adipogenesis and potentially for development of white adipose tissue [43], suggesting that miR-183 may exert an impact on adipogenesis through targeting *LRP6*.

In conclusion, we have identified 75 known miRNAs and 140 novel miRNAs in backfat tissue from Large White and Meishan female pigs. The study expands the repertoire of porcine miRNAs and could be contributive to various and further studies on fat deposition and adipogenesis of pigs. In addition, it indicated that several potential miRNAs may influence adipogenesis and fat deposition. However, whether these miRNAs we have identified also expressed in male pigs or other tissues, organs and whether the potential miRNAs really function in adipogenesis and fat deposition remain to be investigated.

## Materials and Methods

### Ethics statement

All study involving animals were conducted according to the regulation (No. 5 proclaim of the Standing Committee of Hubei People's Congress) approved by the Standing Committee of Hubei People's Congress, P. R. China. Sample collection was approved by the ethics committee of Huazhong Agricultural University. The approved permit number for this study is “No. 30700571”. Animals were allowed access to feed and water ad libitum under same normal conditions and were humanely sacrificed as necessary to ameliorate suffering.

### Animal and sample collection

Six purebred female pigs (3 Large White and 3 Meishan pigs, 150-day-old) provided by Jingpin Pig Station of National Engineering Research Center for Livestock (Huazhong Agricultural University) were used in the present study. The backfat tissues were harvested and immediately frozen in liquid nitrogen, and then kept at −80°C until subsequent analysis.

### Preparation of small RNA library and sequencing

Backfat at the 11–12th rib of 150-day-old Large White and Meishan pigs were collected for RNA isolation. Total RNA was isolated using Trizol agent (Invitrogen), according to the manufacturer's instructions. A 1% agarose gel, stained by ethidium bromide, was run to preliminarily indicate the integrity of the RNA. All RNA samples were quantified and examined for protein contamination (A260 nm/A280 nm ratios) and reagent contamination (A260 nm/A230 nm ratios) by a Nanodrop ND 1000 spectrophotometer.

In this study, two miRNA libraries were constructed, total RNAs extracted respectively from three Large White and Meishan pigs were pooled. Total RNA were prepared for small RNA Sequencing-by-Synthesis according to the procedure and standards of the Illumina Sample Preparation Protocol.

### Sequencing data analysis

The raw sequence reads were generated by the Illumina Genome Analyzer at BGI-Shenzhen, China.

In order to determine conserved miRNAs, the filtered sequences were initially used to search the miRBase 15.0 with BLASTN, which allowed a maximum of two mismatches, and the gaps were counted as mismatches. The criterion was then implemented according to reported miRNA protocol [44].

Potentially novel miRNAs were identified by folding the flanking genome sequence of unique small RNAs using MIREAP( https://sourceforge.net/projects/mireap/).

Comparison of the known miRNA expression between two samples was conducted to find out the differentially expressed miRNAs. The expression of miRNA was shown in two samples by plotting Log2-ratio figure and Scatter Plot. The procedures are shown as below: (1) Normalize the expression of miRNA in two samples (backfat of Large White and Meishan) to get the expression of transcript per million. When the normalized expression of a certain miRNA was zero between two samples, we revised its expression value to 0.01. While if the normalized expression of a certain miRNA was lower than 1, further differential expression analysis was conducted without this miRNA. Normalized expression (NE) = Actual miRNA count/Total count of clean reads×1000000. (2) Calculate fold-change and P-value from the normalized expression. Then generate the log2ratio plot and scatter plot.

Fold_change = log2 (backfat of Meishan-NE/backfat of Large White-NE).

P-value formula:
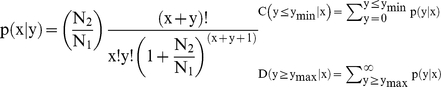



The x and y represent normalized expression level, and the N1 and N2 represent total count of clean reads of a given miRNA in small RNA library of backfat of Large White and Meishan pigs, respectively [32].

### miRNA validation via stem-loop qPCR

Stem-loop qPCR method [27] was used to validate the conserved and novel miRNAs. For each miRNA, three biological replicates were performed. Briefly, the assay was performed using stem-loop RT-PCR followed by SYBR Green Real-time PCR analysis. The ΔΔCt method was used to determine the expression level differences between samples [45]. The significant level was set to 0.05. For the RT-PCR, we adopted the RevertAid™ first strand cDNA synthesis kit (Fermentas, K1622), according to the manufacturer's instructions. Porcine U6 snRNA was used as an internal control and all reactions were run in triplicate. The mature miRNA and primer sequences are available in Table S8.

### miRNA targets predicted by bioinformatics method

To explore the potential function of miRNAs with significantly differential expression in porcine backfat tissue, the targets of miR-183 were predicted by algorithms RNA22, RNAhybrid, TargetScan, DIANA LAB and PicTar [46]–[50].

## Supporting Information

Figure S1 **The work flow of Solexa sequencing.** A. The experiment process. B. The whole data analysis process.
(TIF)

Table S1 **The detailed sequence information in Large White and Meishan small libraries.**
(XLS)

Table S2 **Porcine miRNAs identified using Solexa sequencing.**
(XLS)

Table S3 **Detailed information of known porcine miRNAs in Large White and Meishan small libraries.**
(XLS)

Table S4 **The sequences and secondary structures of predicted novel miRNAs in Large White and Meishan small libraries.**
(XLS)

Table S5 **Single nucleotide substitution in seed region in both small RNA libraries.**
(XLS)

Table S6 **Clustered miRNAs in porcine backfat tissue.**
(XLS)

Table S7 **Conserved porcine miRNAs from vertebrates to invertebrates.**
(XLS)

Table S8 **The mature miRNA sequences and primer sequences of stem-loop qPCR.**
(XLS)
