# Supplementary figures and images for: Solexa Sequencing Identification of Conserved and Novel microRNAs in Backfat of Large White and Chinese Meishan Pigs
Source: PLoS One. 2012 Feb 15;7(2):e31426. doi: 10.1371/journal.pone.0031426 (PMC3280305; doi:10.1371/journal.pone.0031426)

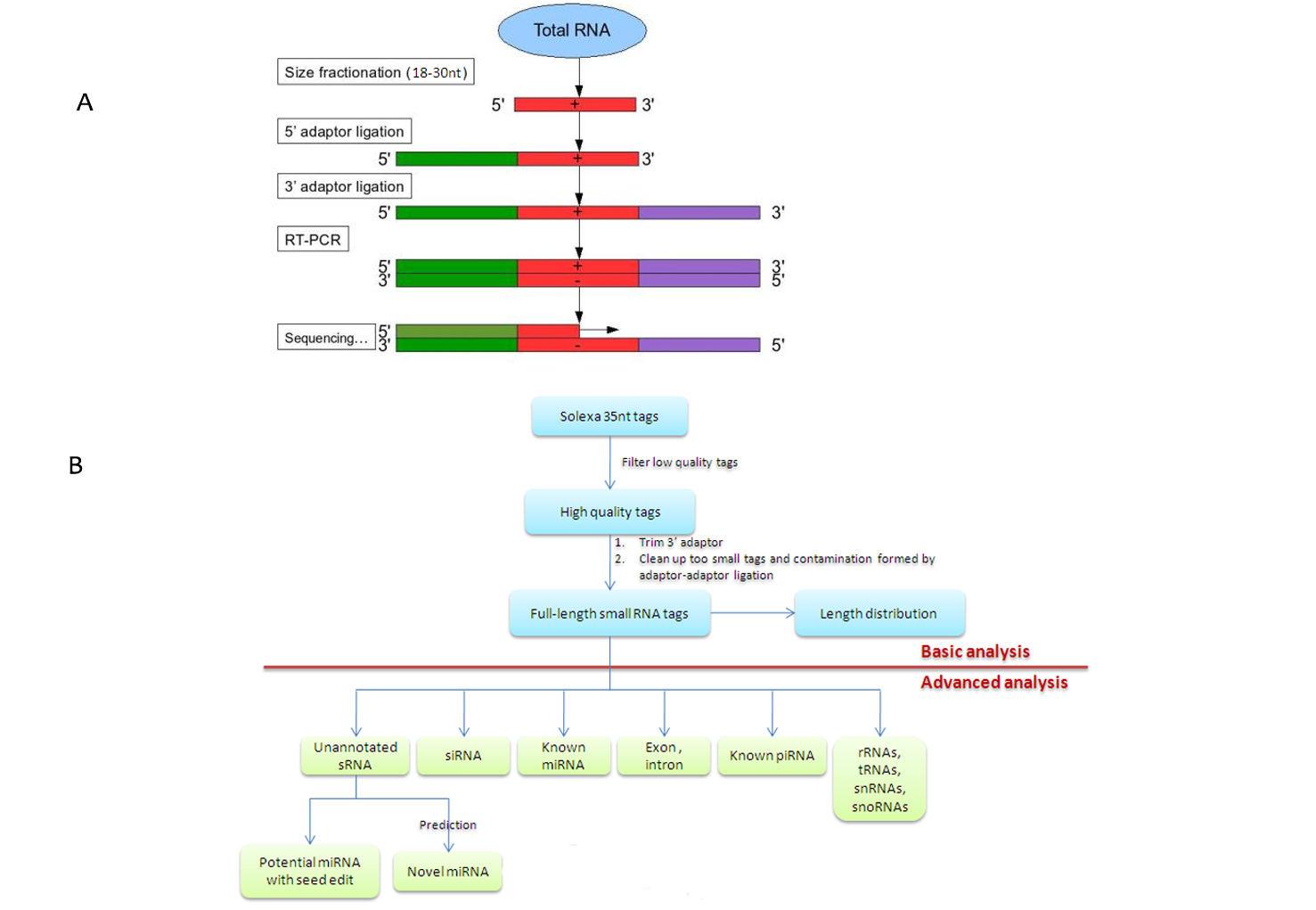

Supplement: Figure S1 — The work flow of Solexa sequencing. A. The experiment process. B. The whole data analysis process. (TIF) [file pone.0031426.s001.tif]
